# Chronic kidney disease and valvular heart disease: State of the art

**DOI:** 10.14814/phy2.70544

**Published:** 2025-09-21

**Authors:** S. Saltarocchi, M. D'Abramo, P. De Orchi, F. Spunticchia, M. Totaro, E. M. Cirio, F. Miraldi

**Affiliations:** ^1^ Division of Cardiac Surgery Azienda di Rilievo Nazionale e Alta Specializzazione “G. Brotzu” Cagliari Italy; ^2^ Internal, Clinical, Anesthesiological and Cardiovascular Sciences Department Sapienza University of Rome Rome Italy

**Keywords:** calcification, chronic kidney disease, inflammation, MAC, valvular heart disease

## Abstract

Chronic kidney disease (CKD) and valvular heart disease (VHD) frequently coexist and are associated with a significant increase in morbidity and mortality. Their interplay is complex and multifactorial, involving shared pathophysiological mechanisms such as chronic inflammation, mineral and bone disorder, vascular and valvular calcification, and neurohormonal activation. These factors contribute to a bidirectional relationship in which each condition can exacerbate the progression and clinical consequences of the other. This review provides a comprehensive synthesis of the current evidence on the epidemiology, pathogenesis, and clinical impact of the CKD–VHD association. Special attention is given to the mechanisms underlying valvular calcification in the uremic milieu, the diagnostic challenges posed by overlapping symptoms, and the prognostic implications of valvular disease in patients with impaired renal function. Furthermore, this paper critically examines the available therapeutic options, including medical management, surgical and transcatheter interventions, and their outcomes in CKD patients. Given the limited evidence from randomized controlled trials in this population, our work also identifies key knowledge gaps and highlights future research directions, advocating for multidisciplinary approaches and tailored strategies. A better understanding of this cardio‐renal interaction is crucial to optimize clinical decision‐making and improve patient outcomes.

## 1 INTRODUCTION

Chronic kidney disease (CKD) is a global health burden that is frequently associated with cardiovascular disease (CVD) and, more specifically, with valvular heart disease (VHD). CKD is described as any abnormality of kidney structure or function present for at least 3 months with implications for health, as defined by an estimated glomerular filtration rate (GFR) <60 mL/min/1.73 m^2^ or by markers of kidney damage (Kipourou et al., 2022; Official Journal of the International Society of Nephrology KDIGO, 2012). Valvular heart pathology, mainly left‐sided, is a common finding in patients suffering from chronic renal failure, particularly in those with end‐stage renal disease (ESRD) and on hemodialysis (HD) (Hoevelmann et al., 2021; Kipourou et al., 2022). Moreover, it is acknowledged that worsening of VHD and progression to heart failure (HF) is faster in these patients than in the general population (Hoevelmann et al., 2021; Kipourou et al., 2022; Marwick et al., 2019; Ternacle et al., 2019). At the base of this so‐called cardiorenal syndrome—where it is often difficult to determine whether cardiac or renal dysfunction is the primary driver (Ronco et al., 2010)—lies a combination of pathophysiological factors. These include a state of volume overload, which induces left ventricular (LV) dilation and remodeling, arterial hypertension, often secondary to systemic vascular calcification, and a dysregulation of bone metabolism and calcium homeostasis that contributes to valvular calcification and leaflet thickening (Marwick et al., 2019; Ternacle et al., 2019). For the same reasons, deterioration of bioprosthetic valvular tissue seems to be accelerated in this subset of patients (Takemura, 2013; Ternacle et al., 2019). CVD in renal failure patients occasionally emerges also through arrhythmias or through the development of infective endocarditis, with dreadful consequences (Hoevelmann et al., 2021; Ludvigsen et al., 2016; Ternacle et al., 2019).

This review aims to summarize the most important aspects of VHD in CKD patients, ranging from epidemiology and pathophysiology to future preventive measures and current therapeutic perspectives.

## 2 EPIDEMIOLOGY AND RISK FACTORS

Chronic kidney disease is a progressive, irreversible clinical condition that affects approximately 10% of individuals worldwide, whose prevalence rises in the elderly, reaching 15%–40% in people aged over 65 (Carney, 2020; Kipourou et al., 2022; Marwick et al., 2019; Villain et al., 2020). Patients affected by CKD are at increased risk of developing CVD of all kinds and, specifically, are more likely to die because of a cardiovascular cause than for renal failure per se (Hoevelmann et al., 2021; Kipourou et al., 2022; Ternacle et al., 2019).

Valvular heart disease is highly prevalent in CKD patients, mainly due to the formation of valvular calcifications (VC) which generate stenosis and/or regurgitation and which are apparently eight times more present in patients with ESRD (Hoevelmann et al., 2021; Ureña‐Torres et al., 2021) and three times more present in patients with CKD in general (Kipourou et al., 2022). Furthermore, the process of VC is accelerated in ESRD patients, occurring one to two decades earlier than its usual course (Hoevelmann et al., 2021; Kipourou et al., 2022; Marwick et al., 2019; Ternacle et al., 2019; Ureña‐Torres et al., 2021) and correlating with higher cardiovascular and all‐cause mortality risk in dialysis patients (Hoevelmann et al., 2021; Paoletti et al., 2016; Wang et al., 2018). The reduction in aortic valve area was estimated to progress at about 0.2 cm^2^ per year in CKD patients, compared to the 0.1 cm^2^ per year of non‐CKD patients (Marwick et al., 2019; Perkovic et al., 2003).

As reported by several studies on the subject, the prevalence of VC in CKD varies between 23% and 84% for the aortic valve and between 5% and 59% for the mitral valve (Kipourou et al., 2022; Marwick et al., 2019; Ternacle et al., 2019); the presence of VC in dialysis patients ranges between 28% and 55% for the AV and between 25% and 59% for the MV (Braun et al., 1996; Hensen et al., 2018; Hoevelmann et al., 2021; Kipourou et al., 2022; Mansur et al., 2020; Matsuo et al., 2018; Ternacle et al., 2019; Ureña‐Torres et al., 2021). Moreover, with worsening eGFR, AV calcification increases (Guerraty et al., 2015; Marwick et al., 2019). On the other hand, the prevalence of VHD in patients with CKD seems to be around 14%, twice that of the general population (7%) (Marwick et al., 2019; Saran et al., 2018). More specifically, Samad et al. (Samad et al., 2017) revealed that: at least mild aortic stenosis (AS) was present in 9.5% of patients compared to 3.5% of patients without CKD; at least mild mitral regurgitation (MR) was present in 42.9% of patients compared to 23.8% of non‐CKD patients; at least mild mitral stenosis (MS) was present in 2.2% of patients vs. 1.1% without CKD; at least mild aortic regurgitation (AR) was present in 19.9% of patients with CKD compared to 10.1% of non‐CKD patients (Marwick et al., 2019; Samad et al., 2017). Even after adjusting these findings for various comorbidities and risk factors, patients with chronic renal failure had higher odds of valvular pathologies, especially those in hemodialysis (Marwick et al., 2019; Samad et al., 2017).

Few studies exist about right‐sided VHD and CKD. In 2017, Zhang et al. (2020) analyzed tricuspid jets of about 500 patients with ESRD: a tricuspid jet was present in 62.6% of patients, 13.9% of whom had at least moderate entity. Scarce evidence exists, instead, about primary pulmonary valve involvement in CKD, whereas secondary pulmonary regurgitation related to pulmonary hypertension is relatively common in ESRD patients.

## 3 ETIOLOGY AND PATHOPHYSIOLOGY

### 3.1 Molecular and cellular mechanisms

#### 3.1.1 Inflammation

Valvular heart disease in chronic kidney disease is a complex multifactorial entity still incompletely understood. However, the molecular and pathogenetic mechanisms that characterize this pathology are similar to those characterizing atherosclerotic disease (Hoevelmann et al., 2021; Romiti et al., 2023; Ternacle et al., 2019). Inflammatory changes are thought to play a decisive role in the development of VHD in CKD and, more specifically, in the formation of valvular calcifications through multiple mechanisms that involve endothelial dysfunction, lipid infiltration, and oxidative stress (Hoevelmann et al., 2021; Ternacle et al., 2019; Ureña‐Torres et al., 2021). These alterations act not in isolation, but in a synergistic and self‐sustaining fashion, where each pathway contributes to and reinforces the others. The release of proinflammatory cytokines—triggered by both mechanical and metabolic stress—promotes immune cell recruitment and matrix remodeling, setting off a vicious cycle of valvular injury and maladaptive tissue responses (Ternacle et al., 2019). Uremic inflammation, typical of ESRD, exacerbates this background, accelerating atherogenesis and calcification. Notably, levels of C‐reactive protein are typically elevated in these patients and correlate with disease severity.

#### 3.1.2 Endothelial dysfunction

An important early feature characterizing valvular leaflet modifications in CKD is endothelial dysfunction, which, by disrupting the basement cellular membrane, enhances the deposition of lipids and inflammatory cells in the valvular tissue, probably due to the augmented turbulence in blood flow that is typical of these individuals (Hoevelmann et al., 2021; Ternacle et al., 2019).

Apart from being increasingly exposed to cardiovascular comorbidities (i.e., hypertension, dyslipidemia, diabetes and atherosclerosis) which per se induce vascular stiffening and endothelial injury, patients affected by CKD typically suffer from fluid imbalance, anemia, and arteriovenous shunts across fistulae that determine a state of hemodynamic overload and high cardiac output, leading to increased flow velocity, increased flow turbulence, especially across the aortic valve, and subsequent shear stress and endothelial damage. As a result, the barrier function of the endothelial layer is progressively loosened, facilitating lipid and inflammatory/immune cell deposits and causing the activation of the coagulation cascade, leukocyte adhesion, and smooth muscle cell proliferation (Marwick et al., 2019; Ternacle et al., 2019). These changes have been extensively investigated in the aortic valve apparatus, which is composed of two populations of cells: AVEC (aortic valve endothelial cells) and AVIC (aortic valve interstitial cells) (Otto et al., 1994; Romiti et al., 2023). The process of aortic valve calcification occurs in two steps: an initiation phase, driven by the inflammatory response, and a propagation phase, in which the calcific pathways seem to predominate in disease progression (Otto et al., 1994; Romiti et al., 2023; Yi, 2018).

**FIGURE 1:**
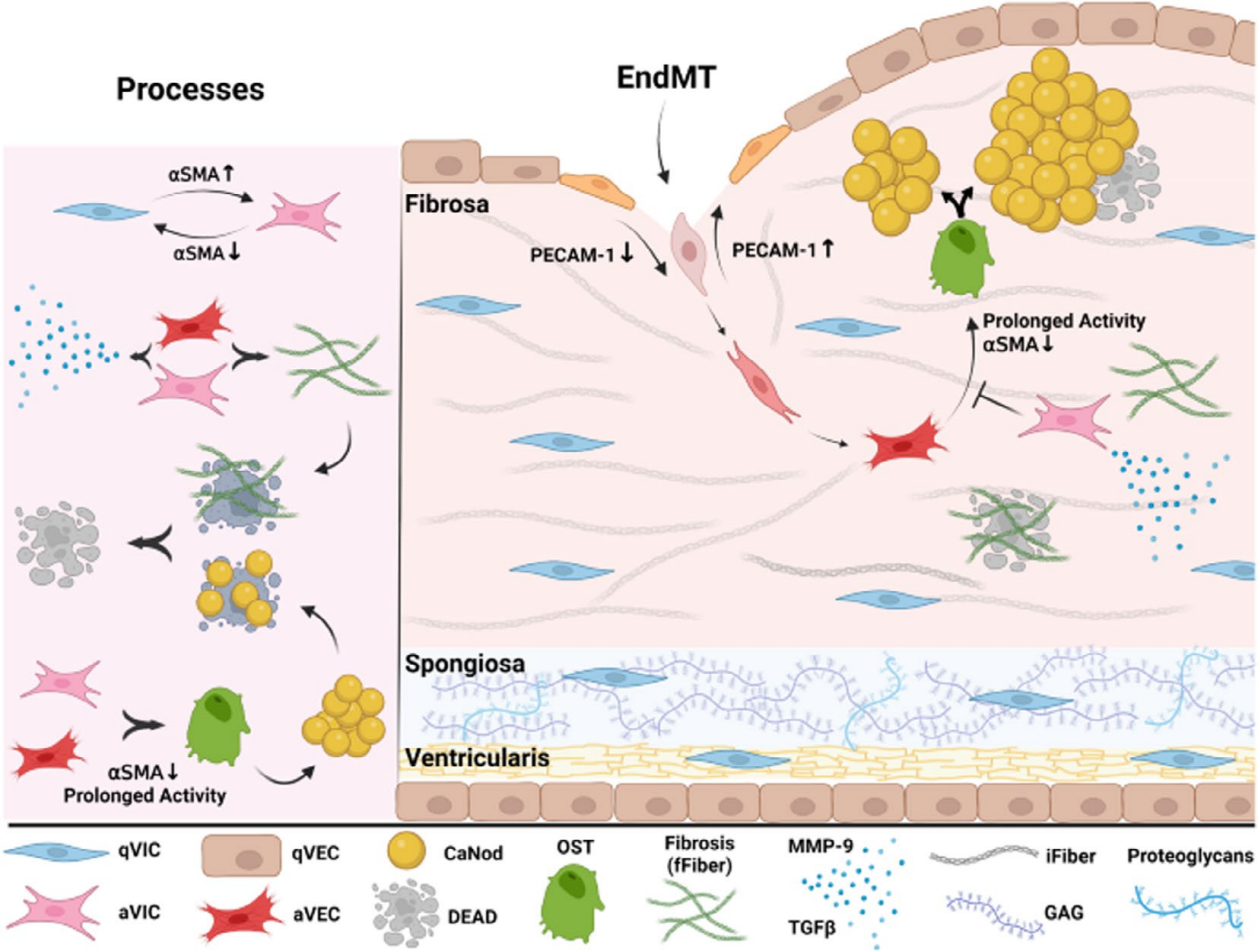
Calcification model of the AV leaflets. The fibrosa is the outermost layer of the valve, covered by qVECs and primarily composed of collagen fibers. The spongiosa is the middle layer, mainly composed of proteoglycans and GAGs. The ventricularis is the innermost layer of the valve, predominantly composed of elastic fibers and covered by qVECs. qVECs can undergo endothelial‐to‐mesenchymal transition upon adhesion molecules downregulation, becoming AVECs, while qVICs can be activated by upregulating smooth muscle protein (aSMA), becoming AVICs. Both secrete MMP‐9 and TGFβ and deposit fibers during the fibrosis process. Prolonged activity and downregulation of AVECs and AVICs can lead to differentiation into OSTs, resulting in calcium nodule nucleation and growth. Cells death can derive from entrapment in highly fibrotic or calcified areas, and the resulting dead bodies can act as feeding sites for CaNods. AV, aortic valve; qVECs, valvular endothelial cells; GAGs, glycosaminoglycans; qVICs, valvular interstitial cells; MMP‐9, matrix metalloproteinase‐9; TGFβ, transforming growth‐factor β. Picture derived from article “Multiscale computational modeling of aortic valve calcification” by Azimi‐Boulali et al. (2024). Reprinted with permission.

Dysregulation of the endothelium starts the first phase and triggers an upregulation of specific adhesion molecules (ICAM‐1 and VCAM‐1) that promote T lymphocyte and macrophage invasion in the aortic valvular fibrosa, inducers of extracellular matrix remodeling and degradation (Otto et al., 1994; Bischoff & Aikawa, 2011; Romiti et al., 2023). Concomitant activation of the immune system leads to cytokine release, namely TNF‐α, TGF‐β1, and IL‐1β, that seems to be employed in the process of valvular calcification (Romiti et al., 2023; Yi, 2018). Importantly, apart from their role in maintaining and exacerbating inflammation through the production of matrix metalloproteinases that regulate apoptosis, cell proliferation, and differentiation, these cytokines are involved in endothelial‐mesenchymal transition of AVEC into AVIC and in inducing their activation in the osteogenic phenotype through different signaling pathways (Bischoff & Aikawa, 2011; Romiti et al., 2023). A schematic representation is offered in Figure 1.

#### 3.1.3 Lipid infiltration

Alteration in lipid metabolism is crucial in the development of VC: disruption of the endothelial barrier layer seems to facilitate lipid invasion in the valvular leaflets and arterial wall, enhancing local inflammation and cellular differentiation pathways (Romiti et al., 2023; Ternacle et al., 2019). Moreover, inflammatory cytokines are thought to increase oxidative stress, favoring the formation of oxidized low‐density lipoproteins and oxidized phospholipids, usually absent in normal valves, which further worsen endothelial dysfunction and inflammation, exacerbating this process and upregulating adhesion molecules involved in the recruitment of immune cells that break down the ECM of the fibrosa layer of the valve (Otto et al., 1994; Romiti et al., 2023; Ternacle et al., 2019).

In parallel, dyslipidemia is per se a risk factor for both aortic and mitral calcific pathologies: it has a role in the development of Mitral Anular Calcification (MAC) and increased levels of LDL or lipoprotein A are involved in calcific aortic valve initiation and progression (Capoulade et al., 2018; Ternacle et al., 2019); moreover, upregulation of LDL receptor‐related protein 5 is linked to skeletal bone development and differentiation of valvular myofibroblast into osteoblasts, starting the propagation phase (Romiti et al., 2023; Ternacle et al., 2019).

#### 3.1.4 Oxidative stress

Oxidative stress plays a pivotal amplifying role across the inflammatory and calcific spectrum of valvular damage. Increased superoxide and hydrogen peroxide levels, coupled with impaired antioxidant defenses, are consistently found in calcified aortic valve lesions (Romiti et al., 2023; Ternacle et al., 2019). These reactive species not only sustain inflammation but also drive phenotypic transformation of AVICs from quiescent to osteogenic and myofibroblastic states (Romiti et al., 2023). A key molecular contributor in CKD is asymmetric dimethylarginine (ADMA), an endogenous inhibitor of nitric oxide synthase. Elevated ADMA impairs nitric oxide (NO) availability, promotes nitric oxide synthase uncoupling, and leads to increased production of reactive oxygen species (ROS) (Ternacle et al., 2019). The resulting oxidative environment contributes to vascular and valvular stiffness, calcification, and increased cardiovascular risk. Importantly, oxidative stress links inflammation and mineral dysregulation, functioning as a biochemical bridge between immune activation and osteogenic transformation in CKD‐related VHD.

## 4 MINERAL METABOLISM AND HORMONAL CHANGES

Other than expressing several osteoblastic markers, explanted stenotic aortic valves have shown histologically microscopical and macroscopical extracellular mineralization, supposedly mediated by cell death and subsequent release of apoptotic bodies, similar to the matrix vesicles found in bone (Ternacle et al., 2019). Mineral and metabolic abnormalities are a feature of ESRD patients with valvular heart disease and seem to affect the pathophysiology of valvular calcifications even in early stages of renal failure, even though the underlying mechanisms are still unclear. Chronic kidney disease is related to Vitamin D deficiency that causes hypocalcemia and hyperphosphatemia which, in turn, induce an increase in parathyroid (PTH) hormone secretion (Ternacle et al., 2019). Although it enhances bone resorption by osteoclastic cells, PTH augmentation also leads to other secondary hyperparathyroidism effects that stimulate ectopic calcifications. This process is often referred to as “calcification paradox” (Ternacle et al., 2019). Moreover, phosphate itself seems able to induce matrix mineralization and activate apoptotic pathways in interstitial cells.

## 5 HAEMODYNAMIC DERANGEMENT

The variation of volume load, characteristic of CKD (particularly HD) patients, can be responsible for creating valvular damage or heart failure. Volume overload can lead to ventricular or atrial enlargement and progressive functional deterioration. Moreover, the additional load caused by the creation of an arteriovenous fistula may cause cardiac decompensation (Marwick et al., 2019; Ternacle et al., 2019). Chamber dilation and fluid imbalance can lead to mitral or tricuspid regurgitation. Mitral disease can therefore be derived from degeneration and calcification, but it can also be a functional insufficiency due to ventricular or atrial remodeling. Tricuspid regurgitation is also affected by volume status. Moreover, the presence of a HD catheter remains an important risk factor for bloodstream infection and subsequent valvular disease (Marwick et al., 2019). In addition, the mechanical stress to which heart valves are subjected (pressure gradient, turbulent flow, high peak blood acceleration and velocities, leaflet vibration through cycling of opening/closure) is accelerated in renal failure patients because of frequent concomitant anemia, AV fistulae, hypertension, high cardiac output state, and volume imbalance (Ureña‐Torres et al., 2021).

## 6 DRUGS

The use of specific medications seems to be associated with the development of VHD in CKD patients. Warfarin accelerates the formation of calcifications not only on heart valves but also on coronary arteries and vessels in general, especially in patients with early‐stage kidney disease (Marwick et al., 2019; Ureña‐Torres et al., 2021). Similarly, calcium supplements and calcium‐based phosphate binders generate a positive calcium balance and hence progression of calcifications, both valvular and coronary (Ureña‐Torres et al., 2021). For this reason, the last KDIGO guidelines recommend diminishing the dosage of these drugs in ESRD patients (Official Journal of the International Society of Nephrology KDIGO, 2012; Ureña‐Torres et al., 2021). Numerous vitaminic supplements that are commonly employed in this setting seem to be also involved: excessive vitamin D assumption can lead to reversible hypercalcemia that could worsen VCs, while valvular tissue calcifies in response to vitamin A therapies, as it induces hypercalcemia as well (Ureña‐Torres et al., 2021).

Among all the mechanisms described, renal disturbances related to mineral and bone disorder (CKD‐MBD) appear to play a particularly significant role (Izzo et al., 2024). Alterations in calcium and phosphate metabolism, secondary hyperparathyroidism, and changes in regulatory factors such as FGF‐23 and Klotho protein have been strongly linked to valvular calcification and disease progression. While inflammation, oxidative stress, and other factors also contribute, current evidence highlights CKD‐MBD as a key driver of valvular pathology in this population. Further studies are needed to clarify the relative impact of these mechanisms and to develop targeted therapies accordingly.

### 6.1 Structural and functional progression

The chronic exposure to the aforementioned molecular insults—inflammation, oxidative stress, endothelial dysfunction, and mineral imbalance—leads to a spectrum of progressive structural changes in the cardiovascular system of CKD patients. These modifications involve both the valves and myocardium and contribute to a bidirectional deterioration of cardiac and renal function, giving rise to a complex cardiorenal syndrome in which the leading cause of multiorgan compromission is not always identifiable.

**FIGURE 2:**
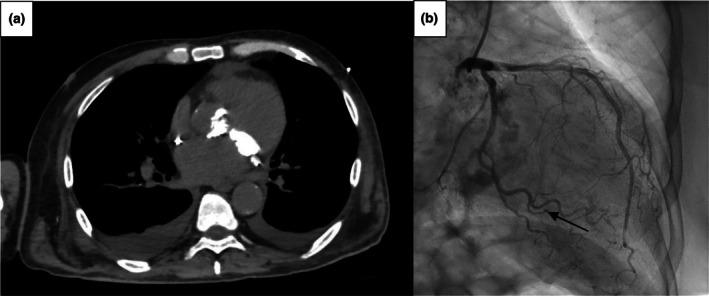
Valvular calcifications and MAC. CT scan showing a case of MAC in axial view, with diffuse calcifications of both the mitral and aortic valve; coronary angiography showing calcification of the mitral anulus (arrow). MAC, mitral annular calcification.

**FIGURE 3:**
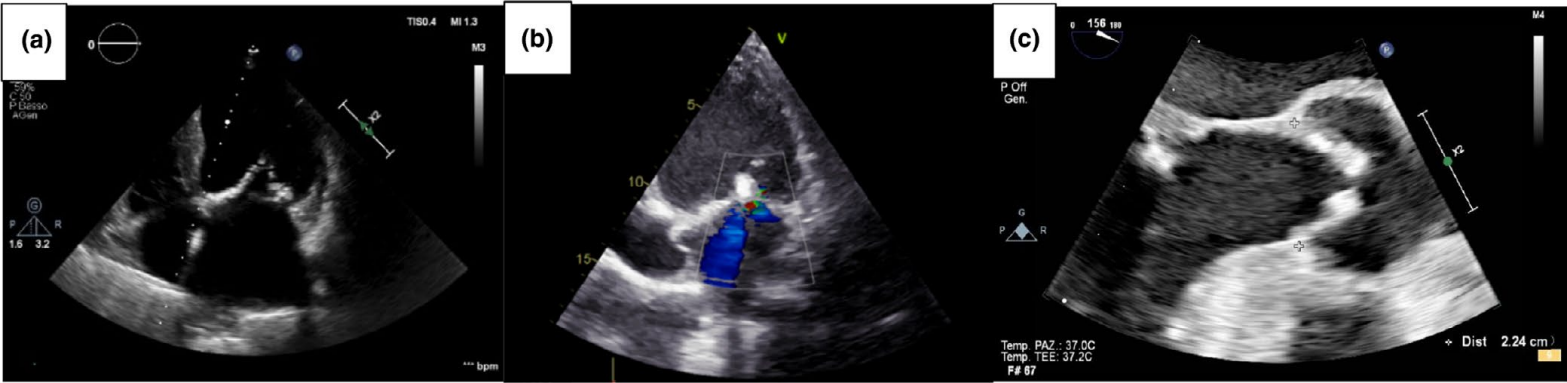
Transthoracic and transesophageal echocardiography. a–b: Apical 2 chamber view showing mitral regurgitation with calcifications of mitral leaflets and anulus (first two panels); c: Enlarged mid‐esophageal long‐axis view emphasizing aortic valve thickening, reduced systolic opening and calcifications in a CKD patient (last panel). CKD, chronic kidney disease.

**FIGURE 4:**
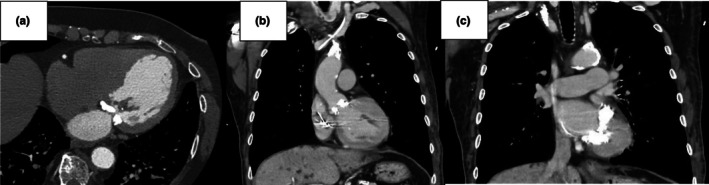
Cardiac CT scan. Cardiac CT showing calcific deposits on the mitral valve in high‐definition images, with involvement of both the anterior and posterior emiannuli (first panel); other cardiac CT images revealing a severe case of MAC, with huge calcifications of the mitral annulus and leaflets but also diffuse calcifications of the aortic valve and innominate artery, with some plaques in the ascending aorta (last two panels).

The left‐sided heart is more commonly involved in renal‐related damage. In general, rapidly progressing valvular pathologies (mainly aortic stenosis) are present in patients with significantly worse renal function (Perkovic et al., 2003). Valvular calcifications, along with fibrosis and thickening, may develop at the level of the aortic cusps and the mitral leaflets (typically at the base), but they can also affect the aortic anulus and the mitral anulus, causing the so‐called mitral annular calcification (MAC), a current challenge in the surgical treatment of the mitral pathology (Figure 2). MAC is a chronic degenerative change that occurs more commonly in the posterior mitral annulus but can affect the entire annular circumference. MAC‐related caseous necrosis and MAC‐related calcified amorphous tumors are lesions characterized, respectively, by liquefaction after calcification of the mitral annulus and agglomeration of calcium deposits, inflammatory cells, and fibrin elements that constitute non‐neoplastic masses (Kipourou et al., 2022). These changes are evident through echocardiography (Figure 3) and cardiac CT scan (Figure 4).

In more advanced cases, calcifications can also affect the subvalvular apparatus or the aortomitral curtain (though this last finding is less common), while the tips of the leaflets and the mitral commissures are less frequently involved (Kipourou et al., 2022). The reduced systo‐dyastolic motion and the generated rigidity and valvular tension can cause considerable valvular pathology in the form of stenosis and regurgitation, or, more commonly, in a mixed fashion. Other than primary/organic forms, valvular regurgitation, mainly mitral and tricuspid insufficiency, can also be functional, due to the increased intravascular volume. Often, in this situation, tricuspid regurgitation develops and, in turn, worsening of mitral regurgitation occurs, or vice versa.

Adaptive mechanisms are often put in place through structural changes and ventricular remodeling. Homeostatic regulation leads to modification of LV geometry, with the development of eccentric and concentric ventricular hypertrophy, leading to myocardial fibrosis and LV remodeling with a reduction in heart function. Added to the increased risk of coronary artery disease and the enhanced secondary pulmonary hypertension, this all leads to a worsening of the picture. In CKD patients, these myocardial modifications start in the early stage of the disease and progress with renal failure but seem to improve after kidney transplantation. The initial changes include myocardial apoptosis and mutation of cardiomyocytes and fibroblasts toward a profibrotic phenotype (Ternacle et al., 2019). These phenomena induce myocardial dysfunction with abnormal global longitudinal strain, increased LV filling pressures, and impaired relaxation. Left heart remodeling through hypertrophy and dilation causes both systolic and diastolic dysfunction, which predicts adverse outcomes (Ternacle et al., 2019).

Moreover, sudden cardiac death (SCD) is the leading cause of cardiac death in patients with ESRD worldwide (Fujii & Joki, 2017). The cause of death is often a fatal tachyarrhythmia, but some SCDs were attributable to bradyarrhythmic episodes. SCD is often related to QT prolongation and torsades de pointes (Fujii & Joki, 2017). While “metastatic” calcifications affecting the heart conduction system could explain fatal bradyarrhythmias through calcifications involving the AV node, causing complete heart block (Fujii & Joki, 2017).

## 7 MEDICAL THERAPY

There are no effective medical therapies able to treat either valvular heart disease or chronic kidney disease. Standard pharmacological management is often required to control the hemodynamic consequences of valvular dysfunction. Diuretics are commonly used to manage volume overload, reduce pulmonary and systemic congestion, and alleviate symptoms of dyspnea, particularly in patients with mitral or tricuspid regurgitation (Vahanian et al., 2022). Vasodilators, including ACE inhibitors and nitrates, may be beneficial in reducing afterload and improving forward flow in selected patients with reduced systolic function or concomitant hypertension, although their use must be balanced against the risk of hypotension and worsening renal perfusion, especially in advanced CKD stages (Vahanian et al., 2022). These agents, however, do not modify the underlying valvular pathology, and their effect remains symptomatic.

Of particular interest in recent years are renin–angiotensin–aldosterone system (RAAS) blockers and statins, though controversial results have been obtained. RAAS blockers seem to slow down VC formation in aortic stenosis, both in human and animal models (Ureña‐Torres et al., 2021). Moreover, due to the shared pathophysiology between atherosclerosis and valvular calcifications, it would be reasonable to consider taking statins for preventing the development of stenotic valvular disease, especially when concomitant renal disease exists. In fact, contradictory findings have emerged, as some studies have reported no important changes while in others statin use has been correlated to increased calcium deposition; however, some authors believe that the inefficacy of lipid‐lowering agents is due to their introduction only in late stages of the disease, when there is already irreversible fibrosis of the leaflets (Romiti et al., 2023; Ureña‐Torres et al., 2021). Moreover, statins cause a potential incrementation of lipoprotein(a) levels; for this reason, inhibitors of PCSK9 currently available in commerce, like the monoclonal antibodies evolocumab and alirocumab seem to reduce the levels of Lp(a) and potentially the evolution of calcific aortic stenosis, as seen in recent studies on multiple patients (Romiti et al., 2023).

Some HD patients treated with sevelamer show a reduced progression (45%) or even a regression (26%) of VC as compared with only 28% or 10% of those treated with calcium‐phosphate binders, and this is the reason why the use of the latter is discouraged by the latest guidelines (Ureña‐Torres et al., 2021; Kidney Disease: Improving Global Outcomes (KDIGO) CKD‐MBD Update Work Group, 2017).

It seems that preserving a normal vitamin D level could help maintain a normal bone turnover and contrast the development of valvular calcifications. In animal models, high‐dose vitamin D supplementation accelerated the progression of AV stenosis, with an increase in calcium‐phosphate products; in parallel, other studies demonstrated that low‐dose vitamin D intake did not have any effect in VC progression (Fujii & Joki, 2017). This could be explained by a supposed biphasic dose–response curve between vitamin D and vascular calcifications, with adverse effects obtained by both excessively high and low vitamin D intake (Fujii & Joki, 2017).

Antiresorptive agents, mainly alendronate, inhibit the expression of osteogenic proteins, suppress osteoclast activity, and have anti‐inflammatory properties, so they could represent potential therapeutic agents that can slow down the VC process. However, recent literature has not shown any result when bisphosphonates were administered to a population of women over 60 years of age, though there is a selection bias due to osteoporosis (Romiti et al., 2023; Ureña‐Torres et al., 2021). In the same setting, denosumab, a monoclonal antibody used against osteoporosis, has demonstrated slower AS progression in vitro (Romiti et al., 2023).

While new oral anticoagulants (NOACs) seem to inhibit AVICs activation and to reduce aortic valve calcification, it appears that vitamin K supplementation could also inhibit VC formation, increasing the challenge and the paradigm regarding the type of anticoagulant administration in cases of ESRD, especially with valvular involvement (Romiti et al., 2023; Ureña‐Torres et al., 2021). Warfarin, a vitamin K antagonist, has been associated with an increased risk of vascular and valvular calcifications due to its inhibition of matrix Gla protein (MGP), a vitamin K‐dependent inhibitor of soft tissue calcification. This interference with the vitamin K cycle promotes osteogenic transdifferentiation of vascular smooth muscle cells and valvular interstitial cells, accelerating calcific processes, particularly in patients already predisposed to mineral metabolism disorders, such as those with CKD (Romiti et al., 2023). In clinical practice, interference with these pathways has not been proven yet.

Lastly, some newer molecules are being evaluated for their potential role in preventing calcifications and VHD progression in renal patients: anti‐inflammatory agents, such as IL‐37 and IL‐38; antioxidants, such as vitamin K2; and calcification inhibitors, like sodium thiosulphate or SNF472 (Romiti et al., 2023; Ureña‐Torres et al., 2021).

Most studies about these novel therapeutic strategies are still ongoing; thus, results are going to be achieved in the near future.

## 8 SURGICAL THERAPY

As no specific indications exist about the management of VHD in CKD patients and literature about precise timing of intervention is missing, the 2021 ESC guidelines about VHD in patients with normal renal function are commonly utilized in this setting (Vahanian et al., 2022). In general, intervention is recommended in symptomatic patients with severe valvular dysfunction, correcting the defect through valve repair, when technically feasible, or replacement through a biological or mechanical prosthesis.

The prognostic value of CKD in patients who undergo valvular surgery has been extensively reported (Lin et al., 2016; Yamamoto et al., 2013) and integrated in risk‐scoring systems such as EuroSCORE II (Nashef et al., 2012) and Society of Thoracic Surgeons (STS) score (Shahian et al., 2018).

Although there are no different indications for valve surgery in CKD patients, and most of them are symptom‐based, some openings may be considered in peculiar patient populations (Metra et al., 2011).

Determining the optimal time for surgery in CKD individuals is extraordinarily important, especially considering the altered life expectancy of those patients compared to that of the general population, and the impact of early valve surgery on effective prognosis: the mortality rates of dialysis‐dependent patients were reported to be 6.6% in Japan, 15.6% in Europe, and 21.7% in the United States at 1 year (Goodkin et al., 2003). As previously stated, patients that have an indication for kidney transplant (KT) often present important valvular calcifications: Kocyigit et al. evidenced VC in almost 84% of patients suitable for KT (Kocyigit et al., 2015), with no evidence of association of valvular calcification with either pre‐KT dialysis or post‐KT duration. No evidence is present on the possibility that progression of VHD may be slower post‐KT compared with pre‐transplant status, but progression of VHD over time is likely; therefore, many will eventually need valve replacement. In a study by Abbott and colleagues, hospitalizations for VHD were lower among patients after KT as compared with before KT (0.68/1000 person‐years and 0.84/1000 person‐years, respectively) (Abbott et al., 2003).

### 8.1 Aortic valve

For the aortic valve, SAVR (surgical aortic valve replacement) is generally indicated in younger patients at low risk for surgery (age ≤75, STS‐PROM Score/EuroSCORE II ≤4%); TAVR is indicated in older patients with aortic stenosis who are at high risk for surgery (age ≥75, STS‐PROM Score/EuroSCORE II ≥4%) (Kipourou et al., 2022; Marwick et al., 2019; Vahanian et al., 2022). In cases of pure aortic regurgitation, TAVR has been utilized but with suboptimal results and a high rate of complications, especially malposition and prosthesis embolization (Poletti et al., 2023). However, since in CKD the pathophysiology of stenosis and regurgitation roughly overlap, it seems that this obstacle can be easily overcome, though no literature exists on the subject.

Open surgery remains a viable option in most patients, although it carries some hazards: structural deterioration is more prevalent in CKD patients (Hoevelmann et al., 2021; Takemura, 2013; Ternacle et al., 2019), as well as complications such as major bleeding, reduced survival, and reoperation (Glaser et al., 2016; Marwick et al., 2019). Throughout the years, there has been much controversy about the type of prosthetic valve in patients with CKD and ESRD, and no consensus has been reached about the most appropriate type of prosthesis to be used. The reported survival rate at 2, 5, and 10 years for bioprostheses was 40%, 14%, and 5%, respectively, and 40%, 15%, and 4% for mechanical prostheses (Brinkman et al., 2002; Herzog et al., 2002; Kaplon et al., 2000; Lucke et al., 1997; Williams et al., 2016). Tendentially, age at operation, and not the type of prosthesis, was found to be predictive of overall mortality (Chan et al., 2006), with no increased evidence of valve‐related complications between mechanical and bioprostheses. Bioprostheses should not be contraindicated in ESRD patients given the observed rarity of accelerated calcification as well as the poor intermediate‐term survival (Chan et al., 2006). Bioprostheses do not require long‐term anticoagulation therapy, and this may be an important advantage over mechanical valves, especially if one takes into consideration the possible dose adjustment issues during chronic care in dialytic patients and the limited life expectancy. On the other hand, the On‐X© mechanical valve could represent a feasible alternative because of its lower anticoagulation requirements, especially in the aortic position (INR 1.5‐2.5) (Puskas et al., 2018), but no research has been developed on this subject.

As for open heart surgery, Smith found CKD to be an independent risk factor in patients who underwent TAVR (Brinkman et al., 2002; Smith et al., 2011), and some authors highlighted that for every 10 mL/min/1.73 m^2^ decrease in estimated GFR, in‐hospital mortality increased by 8.2% (Ferro et al., 2015). A 2019 meta‐analysis by Siontis et al (Siontis George et al., 2019). comparing TAVR with SAVR in 8020 patients showed a lower all‐cause mortality and lower incidence of stroke in individuals submitted to TAVR after 24 months, regardless of their preoperative risk (Hoevelmann et al., 2021; Siontis George et al., 2019). Makki and Lilly (Makki & Lilly, 2018) evaluated through a meta‐analysis the influence of CKD on short (30 days) and long‐term (12 months) mortality in 10,709 patients undergoing TAVR: a CKD stage 4 or 5 was associated with a significant statistical increase in mortality. Regarding the risk of AKI following TAVR or SAVR (Shah, Chaker, et al., 2019), its incidence was significantly lower (7%) compared with that after SAVR (12%), even if the risk of renal failure requiring dialysis after TAVR was not statistically reduced (2.8% vs. 4.1%). There are no long‐term data available regarding possible early degeneration of TAVR in those patients, and no short‐term evidence is present on early degeneration of the TAVR prosthesis compared to the SAVR prosthesis (Popma et al., 2019; Siontis George et al., 2019). However, other than the general augmented risk of permanent pacemaker implantation (Marwick et al., 2019; Smith et al., 2011), TAVR is also associated with increased complications in CKD patients: Allende and colleagues (Allende et al., 2014) found that advanced CKD (Stages 4–5) was an independent predictor of 30‐day major/life‐threatening bleeding and mortality, and late overall cardiovascular and noncardiovascular mortality. Data from a German registry that evaluated 2000 TAVR, with 56 dialytic patients (Schymik et al., 2019), indicate that this population had a higher periprocedural mortality and also a higher 30‐day mortality (10.7% vs. 1.7), along with poor survival at 1 (57.1% vs. 84.2%) and 3 years (26.8% vs. 66.9%) (Schymik et al., 2019). A real comparison between SAVR and TAVR in patients with CKD/ESRD has not yet been performed, but it appears that patients with CKD seem to benefit from the TAVI procedure: a large registry data (Kumar et al., 2018) evidenced, for TAVI procedures, lower in‐hospital mortality, lower rates of AKI, lower incidence of dialysis‐requiring AKI, and postoperative stroke and significantly shorter length of in‐hospital stay compared with SAVR. In summary, the choice between TAVR and SAVR in CKD patients should be tailored according to a combination of surgical risk, anatomical suitability, stage of kidney disease, and expected survival. TAVR may be favored in older patients or those at high surgical risk due to its minimally invasive nature and shorter recovery time, while SAVR might be more appropriate in younger individuals with longer life expectancy or in cases where anatomical limitations preclude percutaneous access. For patients on dialysis or awaiting kidney transplantation, valve choice (bioprosthesis vs. mechanical) and timing of intervention should consider anticoagulation issues, graft eligibility, and the limited data on long‐term valve durability in this population. Moreover, outcomes among patients with KT undergoing TAVR versus open surgical replacement have only been examined in retrospective analyses, with different results. Larger studies are necessary to identify more reliable estimates of outcomes following TAVR in KT recipients.

### 8.2 Mitral valve

Regarding mitral stenosis, the choice between PMC (percutaneous mitral commissurotomy) and mitral valve surgery relies on patients' risk and anatomic characteristics (Kipourou et al., 2022; Vahanian et al., 2022). Mitral valve repair is rarely performed in these patients, due to the presence of extensive calcifications on the anulus and leaflets. Transcatheter edge‐to‐edge mitral valve repair (TEER) via the MitraClip© device is an attractive option in selected candidates. According to research led by Wang et al (Wang et al., 2015), utilization of the device with subsequent reduction in MR and haemodynamic unloading was associated with improved renal function at 1 year in individuals with baseline kidney dysfunction (Marwick et al., 2019; Wang et al., 2015). Conversely, TEER also seems to be associated with poorer long‐term outcomes in the ESRD population, including an augmented 1‐year mortality and hospitalization in such patients (Lo et al., 2018; Marwick et al., 2019). Furthermore, a recent paper evaluating outcomes of TEER with MitraClip© in more than 5000 patients and, specifically, more than 1000 patients affected by CKD (Shah, Villablanca, et al., 2019), outlined a relationship between renal function and all‐cause mortality: in particular, people with an eGFR ≤30 mL/min and dialyzed patients had a 1‐year mortality of 19% and 30%, respectively, compared to 13% of patients with an eGFR ≥60 mL/min (Hoevelmann et al., 2021; Shah, Villablanca, et al., 2019). Moreover, in cases of diffuse mitral annular calcification, transcatheter procedures are becoming a valid alternative to surgery, with TMVR (transcatheter mitral valve replacement) showing acceptable outcomes (Lo et al., 2018; Shah, Villablanca, et al., 2019).

These data underscore a critical gap in expert consensus documents that, at present, do not make specific recommendations regarding follow‐up and management in CKD patients (Otto et al., 2021; Vahanian et al., 2022).

## 9 PROGNOSIS

As CKD represents one of the leading causes of global mortality, these patients are also known to have significantly greater cardiovascular and all‐cause mortality, with cardiovascular diseases being the main cause of death in these cases, rather than ESRD (Kipourou et al., 2022).

Survival is even poorer in patients affected by CKD and VHD. For the aortic valve, the 5‐year survival estimates of patients with mild, moderate, and severe AS and CKD are 40%, 34%, and 42%, respectively, compared with 69%, 54%, and 67% for those without CKD (Samad et al., 2017). For the mitral valve, the 5‐year survival estimates of patients with mild, moderate, and severe MR and CKD were 51%, 38%, and 37%, respectively, compared with 75%, 66%, and 36% for those without CKD (Samad et al., 2017). In parallel, mortality from valvular surgery in this subset of patients is higher than that of the general population (Kodali et al., 2012; Rankin et al., 2006); this information becomes of the utmost importance when we consider that the threshold for cost‐effectiveness of TAVR is set at a median survival of ≥2 years (Marwick et al., 2019; Reynolds et al., 2012). In a 2002 retrospective study of 5858 dialysis‐dependent patients that underwent valvular surgery, in‐hospital mortality was as high as 20.7% (Herzog et al., 2002) while other series report an early mortality of 29.0% (Chan et al., 2006). However, treating the valvular disease, especially aortic stenosis, may influence outcomes in this subset of patients: an analysis of PARTNER 1, PARTNER 2, and PARTNER 2 S3 trials on 5190 patients found that eGFR remained unchanged or improved after TAVR in 89% of patients, probably due to the increased cardiac output and therefore renal perfusion and diuresis as well as decreased renin–angiotensin–aldosterone system activity and sympathetic tone (Cubeddu et al., 2020). Moreover, increased morbidity and mortality have been observed in CKD individuals undergoing mitral valve surgery (either repair or replacement) (Vassileva et al., 2014); in particular, in dialysis‐dependent patients, 30‐day mortality or major morbidity was 40.9%, compared with 15.9% of non‐dialysis patients (Marwick et al., 2019; Vassileva et al., 2014).

## 10 CURRENT PERSPECTIVES AND FUTURE DIRECTIONS

Several knowledge gaps persist in the understanding and management of valvular heart disease in patients with chronic kidney disease. Future research should aim to define optimal timing and modality of valve intervention in this population, especially through randomized controlled trials comparing TAVR and SAVR in advanced CKD and ESRD. Similarly, long‐term durability data of transcatheter prostheses in CKD patients are urgently needed. Despite growing interest, no pharmacological treatment has yet proven effective in halting or reversing valvular heart disease in CKD patients. Future research should clarify the role of RAAS inhibitors and lipid‐lowering agents, given the conflicting data on their impact on valvular calcification. The potential of non‐calcium‐based phosphate binders, vitamin D modulation (with attention to its biphasic effects), and antiresorptive therapies warrants further investigation, especially in targeted subgroups. Future trials should be mechanistically informed and stratified by CKD stage and comorbidities to identify patient‐specific benefits. Moreover, studies focused on the differential impact of CKD etiologies on valvular calcification and disease progression could guide more personalized strategies. The development of specific recommendations for valve selection and management in pre‐ and post‐kidney transplant patients also represents an important unmet need. Finally, better integration of nephrology and cardiology expertise is essential to design research protocols that reflect the complex interplay of cardiac and renal pathophysiology.

## AUTHOR CONTRIBUTIONS

Conceptualization: SS, PDO, FM, EMC; Data curation: SS, MD, FS; Formal analysis: SS, MD, MT; Funding acquisition: not applicable; Investigation: not applicable; Methodology: PDO, SS, MT; Project administration: not applicable; Resources: SS, MD, FS; Software: not applicable; Supervision: MT, FM, EMC; Validation: MT, FM, EMC; Visualization: SS, MD, FS. Writing—original draft: SS, MD, PDO, FS; Writing—review and editing: MT, FM, EMC.

## FUNDING INFORMATION

No funding information provided.

## CONFLICT OF INTEREST STATEMENT

None.

## ETHICS STATEMENT

The research was conducted in accordance with the principles embodied in the Declaration of Helsinki and in accordance with local statutory requirements.
